# Enhancing the ultrasound-assisted hot air drying of cherry through different pretreatments: Effect on drying characteristics, physicochemical quality, microstructure, texture and sensory evaluation

**DOI:** 10.1016/j.ultsonch.2025.107578

**Published:** 2025-09-19

**Authors:** Hongyang Lu, Guojun Ma, Fangxin Wan, Zepeng Zang, Yanrui Xu, Bowen Wu, Lingli Li, Zelin Liu, Xiaopeng Huang, Fei Dai

**Affiliations:** College of Mechanical and Electrical Engineering, Gansu Agricultural University, Lanzhou 730070, China

**Keywords:** Cherry, Different pretreatments, USA-HAD, Drying characteristics, Physicochemical quality

## Abstract

This study aims to optimize the drying performance and quality attributes of cherries through the application of diverse pretreatment techniques. Five pretreatment methods were employed prior to ultrasound-assisted hot air drying (USA-HAD): sodium carbonate and ethyl oleate (SC + EO), ethanol (CA), water blanching (WB), freezing (FZ), and sodium hydroxide (SH). The impact of these pretreatments on drying kinetics, physicochemical properties, microstructural characteristics, and sensory attributes of cherries was thoroughly assessed. The results demonstrated that all pretreatments significantly reduced drying time (16.67∼38.89 %), enhanced drying rates, and notably diminished energy consumption (21.25∼47.17 %). Cherries pretreated with SC + EO and subjected to USA-HAD exhibited markedly elevated (P < 0.05) concentrations of anthocyanins, total phenolic content (TPC), antioxidant activities (DPPH and ABTS), as well as individual sugars, malic acid, and quinic acid. Microstructural analysis revealed that SC + EO pretreatment effectively compromised the waxy cuticle of the cherry skin, promoting the formation of microporous channels, thereby accelerating moisture migration. Sensory evaluation and principal component analysis further corroborated the superior performance of SC + EO-pretreated cherries in terms of color, flavor, texture, and overall acceptability. In conclusion, SC + EO pretreatment proved to be a highly efficacious method in enhancing both drying efficiency and product quality, with substantial potential for industrial application.

## Highlights

• Combined pretreatments reduced cherry drying time by 17-39%.
• Pretreatment technology lowered energy consumption by 21-47%.
• SC + EO pretreatment was the best way to improve quality properties.
• (SC+EO)+USA-HAD drying yielded the highest overall acceptance.
• WB pretreatment destroyed the wax layer to enhance transfer efficiency.

## 1 Introduction

Cherries (Prunus species, Rosaceae family) are nutrient-dense fruits, abundant in anthocyanins, sugars, amino acids, TPC, vitamins, and organic acids. These attributes contribute to their notable antioxidant properties and various health benefits. Cherries are primarily cultivated in North America, Europe, and Asia [1], [2]. However, due to their high moisture content and elevated post-harvest respiration rates, fresh cherries exhibit rapid spoilage, making them highly perishable and difficult to preserve. Furthermore, they are susceptible to microbial contamination during transportation and storage [3], [4]. Consequently, extending the shelf life of cherries to improve their economic value remains a significant challenge in post-harvest processing.

With the growing consumer demand for natural, nutritious, and functional products, dried fruits and vegetables have emerged as a vital category in food processing. These products not only play a key role in extending shelf life but also contribute to enhancing the added value of food. Drying is a pivotal process in the preservation of fruits and vegetables, significantly improving food safety, flavor, and shelf-life. Hot air drying (HAD) remains the most widely used drying method, leveraging heated air to facilitate moisture migration and evaporation through convective heat and mass transfer, thereby achieving dehydration. Due to its low cost, high throughput, and operational simplicity, HAD is extensively utilized across agricultural and food processing industries [5]. However, traditional HAD methods are still hindered by several limitations, including prolonged drying times, high energy consumption, and substantial nutrient degradation [6].

To address the limitations of conventional HAD, researchers have been exploring more efficient approaches that satisfy the dual demands of modern food production-product quality and processing efficiency. Among emerging dehydration techniques, ultrasound-assisted (USA) drying has gained considerable attention due to its ability to enhance heat and mass transfer, reduce drying time, and improve product quality through cavitation, mechanical, and thermal effects [7], [8]. Previous studies have demonstrated the advantages of ultrasound (US)in accelerating water migration, increasing drying rates and enhancing product quality. Yu et al. [9] reported that the application of US significantly reduced both drying time and energy consumption during USA-HAD of camellia seeds. Ren et al. [10] applied US pretreatment during the HAD process of onions, which resulted in the retention of bioactive compounds and enhanced product quality. Tran et al. [11] combined US with HAD to dry carrots, observing increased moisture diffusivity and improved color in the final product. Additionally, Roppolo et al. [12] demonstrated that integrating US into the HAD process effectively preserved the antioxidant activity and phenolic compound content of blackberries.

Notably, the efficiency and quality of the drying process are influenced not only by the applied technology but also by the intrinsic structural characteristics of the fruits and vegetables. The cherry skin, for example, is characterized by a dense waxy layer and keratinized structure, which act as natural barriers during fruit development. However, during the drying process, these features hinder moisture diffusion and heat transfer, leading to reduced drying rates, higher energy consumption and deterioration of product quality. To overcome these challenges, various pretreatment technologies have been extensively utilized to reduce the structural resistance of fruit and vegetable skins, thereby enhancing drying performance and improving the final product quality. Commonly employed pretreatment methods include chemical approaches, such as the use of alkaline solutions, ethyl oleate, ethanol, and similar agents, along with conventional thermal treatments (e.g., blanching, steaming) and physical methods (e.g., freezing). Alkaline solutions effectively dissolve the waxy layer on the surface, increasing cell membrane permeability [13]. Ethanol, with its low boiling point and surface tension, facilitates faster water migration [14]. Ethyl oleate, due to its hydrophobic nature, enhances oxygen isolation, inhibits the degradation of anthocyanins and phenolic compounds, and promotes micropore formation, thereby improving drying efficiency [15]. Blanching and steaming expose the fruit to high temperatures for short durations, inactivating enzymes and disrupting surface tissues to minimize oxidative damage [16]. Freezing pretreatment induces intracellular ice crystal formation, which disrupts cellular structures and facilitates water diffusion, thus enhancing drying efficiency [17].

In recent years, some studies have explored the effects of US pretreatment, edible coatings, and different pretreatment methods on the drying process and quality of cherries. Relevant studies indicate that edible coatings combined with US pretreatment can effectively enhance the retention rates of anthocyanins and phenolic compounds while improving rehydration properties [18]; microwave pretreatment has also been demonstrated to enhance the TPC, antioxidant activity, and water diffusivity of cherries [19]; furthermore, combining colloid-based coatings with US treatment can improve the color and texture of cherries [20]; ultrasonication combined with edible coating also enhances the antioxidant capacity and sensory properties of cherries during infrared drying [21]. However, although these studies have provided valuable insights into cherry drying, most existing research has focused on either single pretreatments or US pretreatment, with a lack of systematic comparison and investigation of the interactions between different pretreatment methods and USA-HAD technology.

In this context, five pretreatment methods (SC + EO, EA, SH, WB, and FZ) were applied in combination with USA-HAD to systematically investigate their effects on drying characteristics, physicochemical quality, microstructure, and sensory evaluation of cherries. This research aims to address gaps in existing studies and provide novel strategies for high-quality drying of small berry fruits.

## 2 Materials and methods

### 2.1 Experimental materials

Fresh cherries (Meizao) used in this study were sourced from commercial orchards in Tianshui City, Gansu Province, China. The initial moisture content was determined to be 83.9 ± 0.5 % (wet basis) according to standard analytical methods [22]. Following harvest, the fruits were stored under controlled refrigeration at 2–4 °C to maintain postharvest quality. Prior to each experiment, uniform cherries free from damage and consistent in size and color were selected as experimental materials. To minimize browning and anthocyanin degradation, cherries were soaked in a 0.5 % citric acid solution for 15 min. Subsequently, the cherries were removed and dried prior to further processing.

### 2.2 Pretreatment procedure

**Fig. 1:**
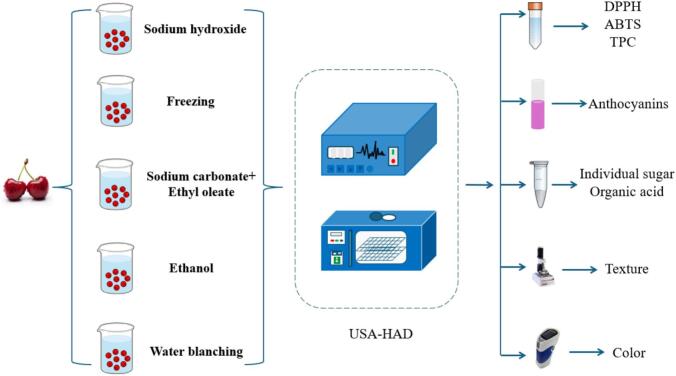
Illustrative diagram of (SC + EO) + USA-HAD, EA + USA-HAD, SH + USA-HAD, WB + USA-HAD, and FZ + USA-HAD treatment of cherry.

The specific pretreatment processes and quality inspection methods for SC + EO, EA, SH, WB, and FZ are shown in Fig. 1.

SC + EO pretreatment: Dissolve sodium carbonate (Na_2_CO_3_) and ethyl oleate in ultrapure water at 25 °C with thorough stirring to prepare a mixed solution containing 2 % Na_2_CO_3_ and 1 % ethyl oleate. Transfer the pretreated cherries into a beaker containing the mixed solution, and soak them for 15 min at 25 °C under dark conditions.

FZ pretreatment: Place the preprocessed cherries into a Leader refrigeration unit (BCD-182LTMPA, Haier Smart Home Co., Ltd., Shandong, China) and freeze at −20 °C for 20 h. Immediately upon removal, submerge the samples in ultrapure water maintained at 25 °C. Thaw the cherries under dark conditions at room temperature (25 °C) for 15 min to partially thaw and equilibrate the surface temperature prior to drying.

WB pretreatment: In accordance with the method outlined by Zhu et al. [23], the pretreated cherries were blanched in ultra-pure water at 100 °C for 2 min. Immediately after blanching, the samples were transferred into ultra-pure water at 4 °C and cooled for 2 min.

EA pretreatment: Prepare a 75 % ethanol solution by mixing anhydrous ethanol with ultrapure water. Transfer the pretreated cherries into a beaker containing the 75 % ethanol solution and soak for 15 min at 25 °C under dark conditions.

SH pretreatment: Prepare a 1 % sodium hydroxide solution by dissolving solid sodium hydroxide powder in ultrapure water at 25 °C under continuous stirring until complete dissolution. Immerse the pre-processed cherries in the prepared solution and soak for 15 min.

Following the aforementioned SC + EO, FZ, EA, WB, and SH pretreatments, gently blot the cherry samples with sterile filter paper to remove residual surface liquid while minimizing disruption to the microscopic structure of the fruit skin. The cherries were longitudinally sliced, and the stones were removed. Subsequently, 200 ± 0.5 g of cherries were accurately weighed. The cherries were arranged with the cut-side facing up in a single, uniform layer on a drying tray, which was then placed in a preheated drying apparatus (YQ101-0A-4A, Beijing Yu Qin Teng Da Pharmaceutical Equipment Co., Ltd., Beijing, China; THD-T1, Dongguan Taiheda Ultrasonic Technology Co., Ltd., Guangdong, China). The parameters for USA-HAD were established based on preliminary tests: hot air temperature of 60 °C, ultrasonic power of 60 W, and ultrasonic frequency of 28 kHz. During the drying process, the weight of the samples was recorded every 30 min using an electronic balance (AUY22, Shimadzu Corporation, Japan) until the moisture content fell below 18 % [24].

### 2.3 Drying rate and moisture ratio

The drying rate and moisture ratio are calculated using formulas (1) and (2).

(1) $$\mathrm{DR}=\frac{M_{t1}-M_{t2}}{t_{2}{-t}_{1}}$$

(2) $$\mathrm{MR}=\frac{M_{t}-M_{e}}{M_{d}-M_{e}}$$

Where *M_t_* is the weight at time t (g/g); *M_e_* is the equilibrium weight (g/g); *M_d_* is the initial weight (g/g); and *M_t1_* and *M_t2_* are the moisture contents at times t_1_ and t_2_ (g/g).

### 2.4 Computation of specific energy consumption

Based on the energy consumption assessment model proposed by Jahanbakhshi et al. [25], the specific energy consumption of the ultrasonic generator and hot air dryer was calculated using Equation (3).

(3) $${{\mathrm{SEC}=SEC}_{\mathrm{hot}}+SEC}_{\mathrm{ult}}$$

Where ${\mathrm{SEC}}_{\mathrm{ult}}$ and ${\mathrm{SEC}}_{\mathrm{hot}}$ are the specific energy consumption of the ultrasonic generator and hot air box, respectively.

### 2.5 Color measurement

Color measurements of the dried cherries were conducted using a color analyzer (CR-410, Konica Minolta, Japan), and the color parameters were calculated in accordance with Equation (4).

(4) $$\Delta E=\sqrt{{\left(L^{*}-L\right)}^{2}+{\left(b^{*}-b\right)}^{2}+{\left(a^{*}-a\right)}^{2}}$$

Where *ΔE* represents the total color change, L*, a* and b* represent the brightness, red-green and blue-yellow values of fresh cherries, and *L*, *a* and *b* represent the brightness, red-green and blue-yellow values of the dried cherries, respectively.

### 2.6 Extraction and determination of anthocyanins

The method of Lu et al. [26] was used to extract and measure the anthocyanins in cherries.

### 2.7 Determination of individual sugars and organic acids

0.500 ± 0.001 g of dried cherry samples were precisely weighed and ground under liquid nitrogen, which were then extracted with 25 mL of 75 % ethanol. The extraction was conducted in a constant-temperature shaker (THZ-98B, Shanghai Boxun Medical Biological Instrument Co., Ltd., Shanghai, China) at 25 °C, 120  rpm in the dark for 48  h. The mixture was then centrifuged at 5500  rpm for 15  min at 4 °C using a centrifuge (TD5Z, Hunan Duoheng Instrument Equipment Co., Ltd., Changsha, China). The supernatant was collected for subsequent analysis of physicochemical properties

### 2.8 Determination of TPC

The TPC of the cherries was determined using the Folin-Ciocalteu reagent method, as described by Xu et al. [27].

### 2.9 Determination of antioxidant activity

The DPPH and ABTS radical scavenging activities of the cherry extracts were determined using a visible light spectrophotometer (V1800, Shanghai Unico Instrument Co., Ltd., Shanghai, China), with minor modifications based on the methods described by Wang et al. [28] and Zang et al. [29]. The detailed procedures were as follows:

DPPH radical scavenging activity: To 3.0 mL of a 10^-4^ mol/L DPPH methanol solution, 60 μL of the extract solution was added. The mixture was thoroughly mixed and shaken in the dark at room temperature for 30 min. The absorbance was then measured at 515 nm, with anhydrous methanol serving as the blank control.

ABTS radical scavenging activity: 35 μL of the extract solution was added to 3.0 mL of the ABTS stock solution. The mixture was thoroughly mixed, shaken for 5 min, and subsequently incubated in a water bath at 30 °C for 30 min. The absorbance was measured at 734 nm, using a 0.1 mol/L phosphate buffer as the control.

### 2.10 Textural characteristics

The texture properties of the cherries were determined using a texture analyzer (TA-XT Plus, Stable Micro Systems, UK) equipped with a P/36 flat-bottom probe. The test conditions were set as follows: a pre-test speed of 2 mm/s, a test speed of 0.5 mm/s and a post-test speed of 2 mm/s, with a compression ratio of 60 %. Each cherry sample was measured at least three times, and the results were averaged.

### 2.11 Microstructure

According to the method of Lu et al. [24], the microstructure of the cherry surface was evaluated using a scanning electron microscope (S3400N, Hitachi, Japan) at 300 × magnification.

### 2.12 Sensory evaluation

Sensory analysis was employed to evaluate the flavor characteristics and overall acceptability of the cherries [30], [31]. Ten individuals, randomly selected from a pool of trained sensory evaluators aged between 18 and 45, participated in the evaluation. A 10-point scale (1-strongly dislike, 10-strongly like) was used to assess the sourness, sweetness, off-odor, color, texture, and overall acceptance of the cherries. To ensure objectivity, all participants tasted the samples with their eyes closed. Each test was performed in triplicate, and the results were averaged.

### 2.13 Statistical analysis

The data were analyzed using the following software: IBM SPSS Statistics 26, Origin 2023 and Microsoft Excel 2016. Significance between different samples was tested using one-way analysis of variance (ANOVA), with *P < 0.05* indicating a significant difference. All experiments were repeated three times, and the results are presented as the mean ± standard deviation.

## 3 Results and discussion

### 3.1 Drying characteristics

**Fig. 2:**
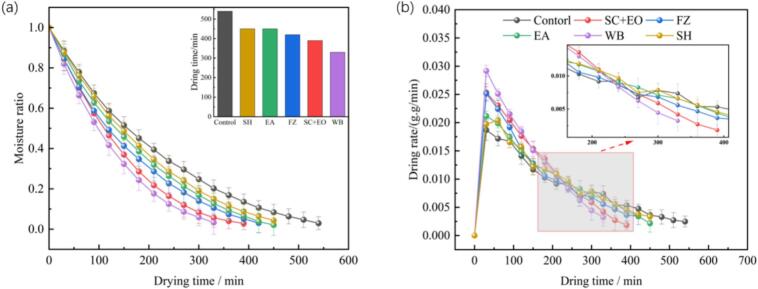
Moisture ratio (a) and drying rate (b) of cherries under different pretreatment conditions.

Fig. 2 presents the drying characteristics curves of cherries subjected to different pretreatment methods. The drying times for cherries treated with SH, EA, FZ, SC + EO, and WB were 450, 450, 420, 390, and 330 min, respectively. Compared to the untreated control group (540 min), these pretreatments shortened the drying time by 16.67 %, 16.67 %, 22.22 %, 27.78 %, and 38.89 %, respectively. Correspondingly, the average drying rates increased by 19.87 %, 19.55 %, 28.53 %, 38.46 %, and 63.46 %. This suggests that the five pretreatment methods effectively reduce cherry drying time and increase the drying rate. Among them, WB pretreatment exhibited the most pronounced effect on the disruption of the surface wax layer, followed by SC + EO. Hot water blanching is known to cause softening and collapse of cellular structures, while elevated temperatures induce protein denaturation in cell membranes, thereby markedly increasing membrane permeability. The wax layer on cherry skin comprises hydrophobic lipids, primarily long-chain fatty acids and their derivatives [32]. In an alkaline ethyl oleate environment, the structure of wax crystals undergoes substantial alteration due to saponification of fatty acids, resulting in gradual dissolution and destruction of the hydrophobic barrier. This process also leads to partial hydrolysis of the cuticle, producing micro-scale cracks and pores, which enhance membrane permeability and facilitate water diffusion and evaporation during drying [33]. During FZ pretreatment, intracellular ice crystals mechanically disrupt the cell membrane and wall, forming microporous channels. The resulting structural damage increases the ease of water diffusion, thereby reducing internal drying resistance [34]. Under EA pretreatment, the low surface tension of ethanol facilitates effective tissue penetration, while the Marangoni effect-driven by surface tension gradients—promotes outward water migration, thus enhancing the drying rate. Furthermore, ultrasound-assisted drying exerts mechanical forces that disrupt the internal tissue matrix, weaken intermolecular bonding, and increase internal water mobility, collectively reducing drying duration [35].

### 3.2 Specific energy consumption (SEC)

**Fig. 3:**
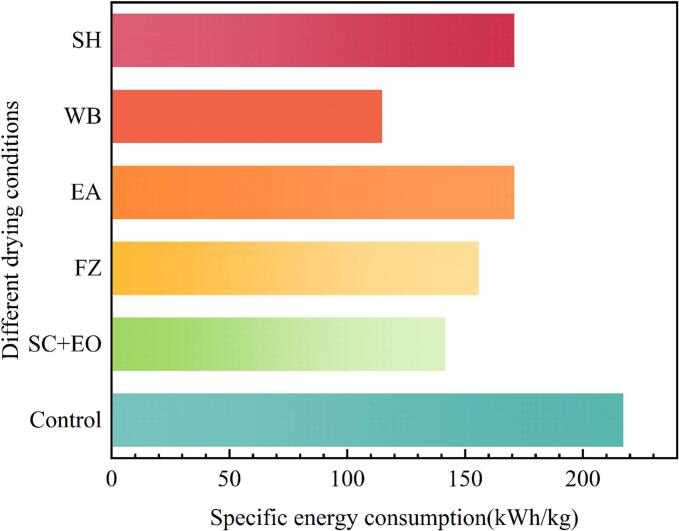
Specific energy consumption of cherries under different pretreatment conditions.

Energy consumption is a critical factor in the drying of fruits and vegetables, as it directly influences drying efficiency, product quality, and resource utilization. Rational control of energy input not only improves drying uniformity and preserves product quality but also reduces production costs while promoting energy conservation and emission reduction. Fig. 3 illustrates the energy consumption of the USA-HAD process for cherries following various pretreatments. Compared with the control group, the SC + EO, FZ, EA, WB, and SH pretreatment groups reduced energy consumption by 34.79 %, 28.20 %, 21.25 %, 47.17 %, and 21.30 %, respectively. These findings demonstrate that combining USA-HAD with pretreatment can effectively reduce energy consumption (21.25∼47.17 %) and thereby lower operational costs. Among the treatments, WB exhibited the greatest energy-saving potential, consuming only 114.76 kWh/kg, followed by SC + EO at 141.57 kWh/kg. The superior performance of WB can be attributed to its ability to reduce drying time and accelerate moisture diffusion. Similarly, SC + EO pretreatment contributed to energy savings and supported environmental sustainability. In conclusion, integrating USA-HAD with appropriate pretreatment strategies holds great potential for minimizing energy consumption during cherry drying.

### 3.3 Color

Color is a crucial quality attribute of fruit and vegetable products, directly influencing consumer purchasing behavior and visual appeal [36]. The effects of various pretreatment methods on the color attributes of cherries are presented in Table 1. The L*, a*, and b* values of fresh cherries are 26.29, 6.26, and 2.30, respectively. Compared to fresh cherries, the brightness values of cherries dried after undergoing different pretreatments were significantly lower. The cherries' surface color darkened, their yellowness values increased, and significant differences in color appearance were observed. This phenomenon may be attributed to the prolonged exposure of cherries to elevated temperatures and humidity during the drying process, which accelerates the degradation of anthocyanins within the cells, ultimately leading to a deterioration in surface quality [5]. Among the five pretreatment methods evaluated, samples treated with WB and SC + EO exhibited the highest L values (26.16 and 25.17, respectively), which were significantly higher than those of the control group and most closely resembled the values of fresh samples. This indicates that these two pretreatment methods are most effective in preserving the cherries' original brightness, likely by inactivating enzymes responsible for browning and forming a protective layer on the surface, thereby reducing pigment degradation during drying. Furthermore, an increase in b values was observed across all dried samples compared to fresh cherries, indicating a general shift toward yellowness. This phenomenon is attributed to the Maillard reaction forming brown compounds or the degradation of chlorophyll. Notably, the SC + EO pretreatment resulted in a b value (2.57) closest to that of fresh cherries, demonstrating its advantage in minimizing yellowness development. However, relative to the control group, the color of the five pretreated cherries was significantly reduced. Among them, cherries pretreated with WB (ΔE = 1.87 ± 0.14) had the smallest color difference, followed by those pretreated with SC + EO (ΔE = 2.55 ± 0.13). This improvement can be ascribed to the protective effects of pretreatment, which mitigate discoloration. Furthermore, pretreatments effectively reduced drying time, curbed the oxidative degradation of bioactive compounds such as anthocyanins and carotenoids, and minimized the occurrence of Maillard reactions and enzymatic browning, thereby preserving the cherries’ original color and overall appearance [37].

**Table 1:** Effect of different pretreatments on cherry color.

Drying conditions	$L^{*}$	$a^{*}$	$b^{*}$	ΔE
Fresh	26.29 ± 0.37^a^	6.26 ± 0.30^b^	2.30 ± 0.21^d^	
Control	19.20 ± 0.39^f^	6.40 ± 0.70^b^	4.06 ± 0.04^ab^	6.36 ± 0.21^a^
EA	23.03 ± 0.25^d^	4.19 ± 1.44^c^	3.31 ± 0.56^bcd^	3.39 ± 0.14^d^
WB	26.16 ± 0.31^a^	6.21 ± 0.71^b^	3.41 ± 0.56^abc^	1.87 ± 0.14^f^
SC + EO	25.17 ± 0.17^b^	6.59 ± 0.57^b^	2.57 ± 0.47^cd^	2.55 ± 0.13^e^
FZ	24.12 ± 0.37^c^	5.07 ± 3.07^b^	3.08 ± 0.60^bcd^	4.06 ± 0.24^c^
SH	20.83 ± 0.25^e^	10.53 ± 0.52^a^	4.41 ± 0.49^a^	5.31 ± 0.15^b^

### 3.4 Anthocyanins

**Fig. 4:**
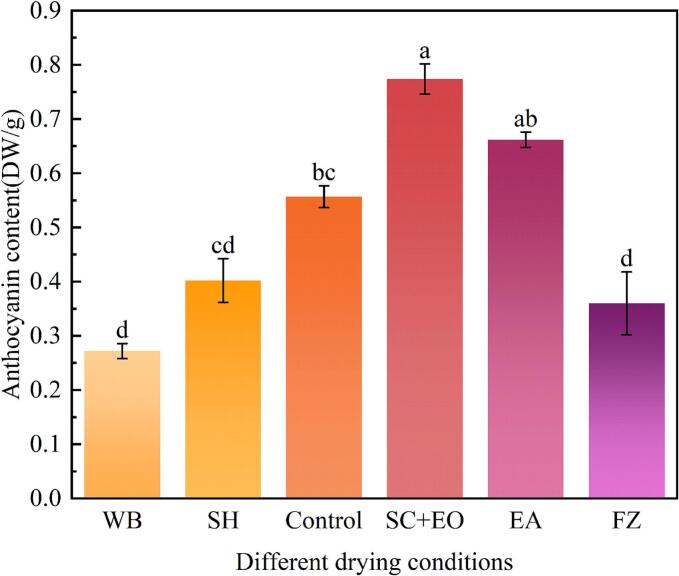
Effect of different pretreatments on the anthocyanin content of cherry blossoms.

Anthocyanin, a natural plant pigment found in cherries, is intimately linked to their color. The elevated content of anthocyanins contributes to the intensified pigmentation of cherries, which positively correlates with consumer preference and purchase intent [38]. As shown in Fig. 4, the anthocyanin content varied significantly among cherries subjected to different pretreatments. The greatest anthocyanin content was detected in the SC + EO group, followed by the EA-treated samples, while the lowest was detected in cherries subjected to WB. This variation may be attributed to the hydrophobic nature of ethyl oleate, which restricts oxygen solubility and subsequently slows anthocyanin degradation. Additionally, the shear force generated by US disrupts cell walls, thereby promoting anthocyanin release [39]. During EA pretreatment, the inhibition of polyphenol oxidase (PPO) activity further reduced enzymatic degradation of anthocyanins [40]. In contrast, the thermal instability of anthocyanins renders them prone to structural breakdown under the elevated temperatures of WB pretreatment, resulting in reduced anthocyanin content. Moreover, the asymmetric collapse of cavitation bubbles and the localized microjets induced by US cavitation generate high internal temperatures, accelerating the thermal degradation of anthocyanins [41]. Another factor contributing to anthocyanin degradation is the formation of hydroxyl radicals from water molecules during ultrasonic cavitation, which cleave the benzene ring structure of anthocyanins [42].

### 3.5 Individual sugars and organic acids

**Fig. 5:**
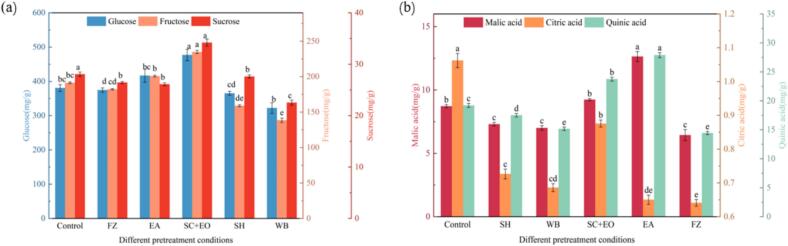
Effect of different pretreatments on the individual sugars and organic acid content of cherries.

The effects of different pretreatments on the contents of individual sugars and organic acids in cherries are presented in Fig. 5. In the control group, the respective contents of glucose, fructose, malic acid, and quinic acid were 380.44, 191.3, 8.72, and 19.21 mg/g. Compared with the control group, the glucose and fructose content of cherries pretreated with SC + EO and EA increased by 25.4 %, 9.4 % and 22.7 %, 4.9 %, respectively. Meanwhile, the levels of malic acid and quinic acid elevated by 5.96 %, 44.95 %, and 23.58 %, 45.13 %, respectively. These enhancements may be attributed to the saponification effect of alkaline ethyl oleate, which disrupts cherry cell walls and facilitates solute diffusion, thereby improving the extractability of sugars and organic acids. Furthermore, ethanol pretreatment is known to inhibit enzymatic activity, thereby reducing the degradation of sugars and organic acids. In contrast, the lowest concentrations of individual sugars were observed in cherries subjected to WB pretreatment. This can be explained by the fact that high-temperature blanching damages the wax layer on the cherry surface, leading to a sharp rise in internal temperature, which intensifies Maillard and caramelization reactions, ultimately reducing the content of individual sugars. Overall, the interactions between sugars and organic acids are antagonistic, with sucrose being irreversibly hydrolyzed into glucose and fructose by invertase activity [29], which accounts for the elevated levels of glucose and fructose, and the relatively low levels of sucrose and organic acids in the cherries.

### 3.6 TPC and antioxidant capacity

**Fig. 6:**
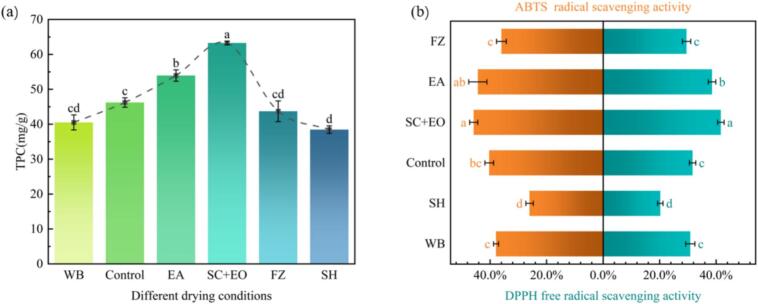
TPC and antioxidant activity of cherries under different pretreatment conditions.

Antioxidant activity mitigates oxidative stress reactions by neutralising free radicals, thereby delaying cell ageing and boosting immunity. Phenolic compounds are commonly used as important indicators for assessing antioxidant capacity due to their reducing power and free radical scavenging ability. As shown in Fig. 6, TPC and antioxidant capacity varied significantly among cherries subjected to different pretreatments. Compared with the control group, cherries pretreated with SC + EO and EA exhibited notable increases in TPC by 36.89 % and 16.72 %, respectively. Correspondingly, DPPH activity increased by 31.54 % and 13.63 %, while ABTS activity rose by 21.76 % and 9.99 %. These results indicate that SC + EO and EA pretreatment effectively preserve the TPC and antioxidant capacity of cherries. This can be attributed to the alkaline ethyl oleate environment, which disrupts cell walls or releases bound phenolic compounds, thus enhancing their extractability and antioxidant capacity. Additionally, an alkaline environment may inhibit the oxidative polymerisation and degradation of phenolic substances. The synergistic effects of ethanol permeation dehydration and ultrasound-enhanced mass transfer further contribute to the retention of phenolic and antioxidant compounds. These findings align with the conclusions of Ren et al. [43] and Osae et al. [44]. In contrast, cherries pretreated with WB and FZ showed marked reductions in TPC and antioxidant activity. This is likely due to the disruption of cell structure caused by WB pretreatment, which accelerates the oxidation of phenolic compounds, leading to substantial degradation of these substances [45]. Similarly, during FZ pretreatment, the formation and growth of ice crystals can rupture cell membranes, resulting in the leakage of phenolic compounds with the exudation of cell sap [46]. Oszmiański et al. [47] reported a comparable decrease in TPC following freezing pretreatment in strawberries. Research shows that antioxidant capacity is positively correlated with TPC, which explains why the antioxidant capacity of cherries decreases after WB and FZ pretreatment.

### 3.7 Texture

Texture is a critical sensory attribute of cherries and is commonly described in terms of hardness, springiness, cohesiveness, gumminess, chewiness, and resilience. Table 2 presents the effects of various pretreatment methods on the textural properties of cherries. Among all treatments, cherries pretreated with SC + EO exhibited superior springiness (86.36 ± 0.02 %), gumminess (16.71 ± 0.97 N), resilience (33.63 ± 0.04 %), and chewiness (13.45 ± 1.46 N). These enhancements may be attributed to the ability of SC + EO to preserve the cellular framework, thereby maintaining structural integrity and improving texture attributes. Compared to the control group, the five pretreatment methods (EA, WB, SE + EO, SH, and FZ) resulted in reductions in the hardness and cohesiveness of dried cherries, with WB and FZ pretreatments showing the most pronounced effects. This can be attributed to the damage of the surface cell structure of cherries during high-temperature blanching, which decreases the hardness and cohesiveness of dried cherries [48], a finding consistent with the conclusions of Zheng et al. [49]. Similarly, during FZ pretreatment, the formation of ice crystals disrupts cellular architecture, leading to mechanical damage and degradation of texture [50]. Zielińska et al. [51] also reported significantly reduced hardness in frozen strawberries. In general, the four pretreatments (EA, WB, SH, and FZ) reduced the textural properties of the cherry samples and also decreased the energy required for chewing the dried cherries before swallowing [52].

**Table 2:** Textural characteristics of cherries after different pretreatments.

Drying conditions	Hardness (N)	Springiness (%)	Cohesiveness	Gumminess (N)	Chewiness (N)	Resilience (%)
Control	25.09 ± 0.86^a^	80.27 ± 0.04^b^	0.72 ± 0.01^ab^	14.01 ± 0.85^b^	11.95 ± 0.62^ab^	29.07 ± 0.05^b^
EA	21.66 ± 0.36^b^	75.68 ± 0.01^cd^	0.80 ± 0.02^a^	13.85 ± 0.15^b^	10.90 ± 0.62a^bc^	28.43 ± 0.07^b^
WB	16.80 ± 0.10^d^	78.30 ± 0.01^c^	0.61 ± 0.03^bc^	10.05 ± 0.09^c^	7.99 ± 0.23^c^	26.42 ± 0.02^bc^
SC + EO	15.81 ± 0.05^d^	86.36 ± 0.02^a^	0.64 ± 0.02^bc^	16.71 ± 0.97^a^	13.45 ± 1.46^a^	33.63 ± 0.04^a^
SH	20.00 ± 0.09^c^	76.39 ± 0.04^c^	0.66 ± 0.07^b^	11.11 ± 1.17^c^	8.84 ± 1.63^bc^	25.59 ± 0.01^c^
FZ	14.18 ± 0.30^e^	72.09 ± 0.02^d^	0.54 ± 0.02^c^	9.17 ± 0.15^c^	10.67 ± 0.26^abc^	25.83 ± 0.01^bc^

### 3.8 Microstructure

**Fig. 7:**
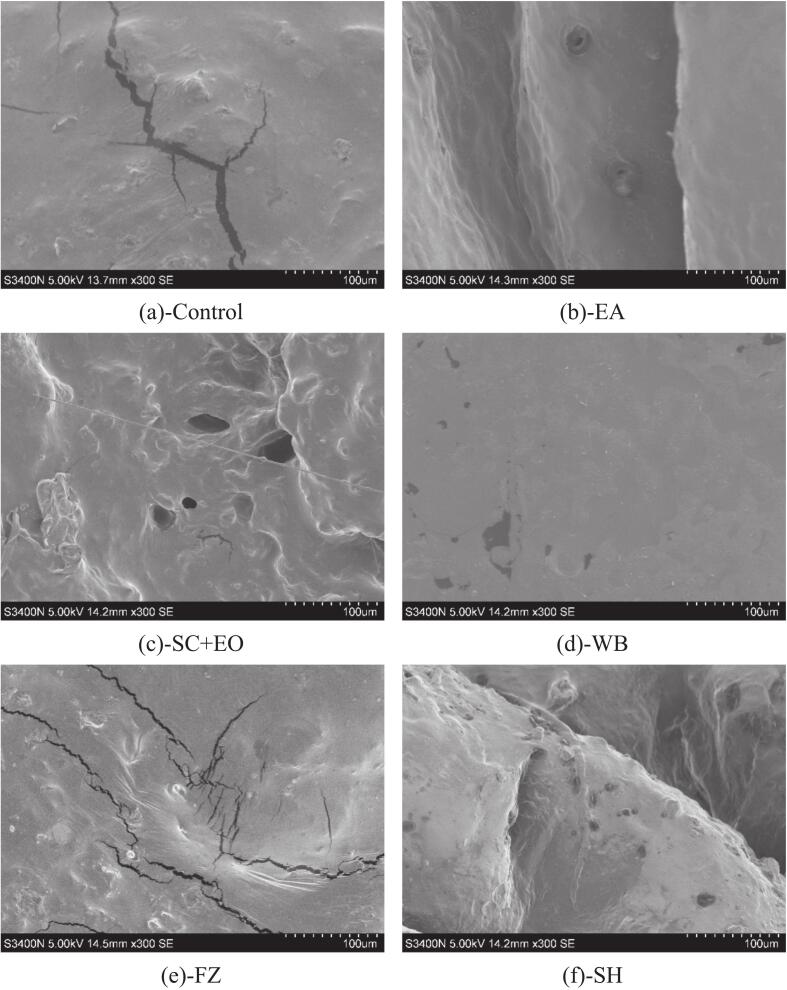
Microstructure of cherries after different pretreatments.

Structural alterations during the drying process critically influence internal moisture diffusion and the efficiency of heat and mass transfer. The microstructural changes in cherries subjected to different pretreatments are illustrated in Fig. 7. All pretreatments induced varying degrees of damage to the waxy cuticle on the cherry surface. Following EA pretreatment, slight wrinkling of the cherry skin was observed, which resulted in the formation of a small number of microporous channels. This is due to the osmotic action of the ethanol solution, which dehydrates and shrinks the cells, causing them to collapse and form wrinkles in the epidermis. Concurrently, the ethanol solution dissolves the wax layer, enhancing the permeability of the epidermis and promoting the formation of microporous channels. Similar microstructural modifications have been reported by Rojas et al. [53] and Feng et al. [54] in ethanol-treated pumpkin and garlic, respectively. After WB pretreatment, the surface of the cherries became smooth, and microporous holes formed. This is likely due to high-temperature blanching, which partially dissolves pectin in the cell walls, while the expansion of intracellular gases under heat promotes cellular collapse and reorganization, resulting in a more homogeneous microporous structure. During the FZ pretreatment process, the growth of ice crystals increases intercellular pressure, which punctures the cell membrane and penetrates the entire cell, leading to extensive internal tissue rupture. This structural damage not only accelerates the diffusion of internal moisture but also enhances heat and mass transfer efficiency.

### 3.9 Sensory evaluation

**Fig. 8:**
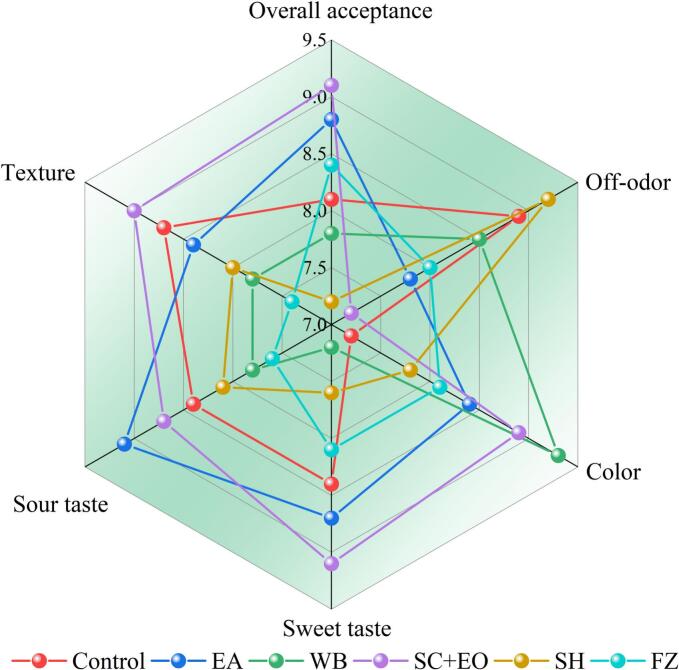
Sensory evaluation of cherries after different pretreatments.

Sensory evaluation plays a crucial role in quality control and consumer acceptance of dried fruit products. Key sensory attributes such as color, aroma, sweetness, sourness, texture, flavor, and overall acceptability serve as direct indicators of both processing efficiency and raw material quality. As shown in Fig. 8, cherries subjected to SC + EO pretreatment received the highest overall acceptability score (9.1), followed by EA (8.8) and FZ (8.4), whereas the SH-treated samples recorded the lowest score (7.2). This clearly indicates that SC + EO-treated cherries are preferred by consumers. Cherries treated with SH had the highest off-odor score (9.2) because residues of the sodium hydroxide solution remained on the surface of the dried samples, resulting in a strong odor. After SC + EO and WB pretreatment, the color of cherries becomes more vivid. This effect can be attributed to the synergistic action of US combined with SC + EO and WB pretreatments, which shortens drying time and mitigates the degradation of anthocyanins and phenolic compounds. Moreover, they helped to delay enzymatic browning and the Maillard reaction, thereby improving the visual appeal of the dried cherries [55]. Volume shrinkage is a critical factor influencing the sensory quality of dried cherries. FZ and WB pretreatment damaged the cell structure due to ice crystal damage or thermal degradation, causing excessive shrinkage during drying. This negatively impacted the chewiness, visual appearance, and textural quality of the cherries, leading to a reduction in their texture scores.

### 3.10 Principal component analysis (PCA) and Cluster analysis (CA)

**Fig. 9:**
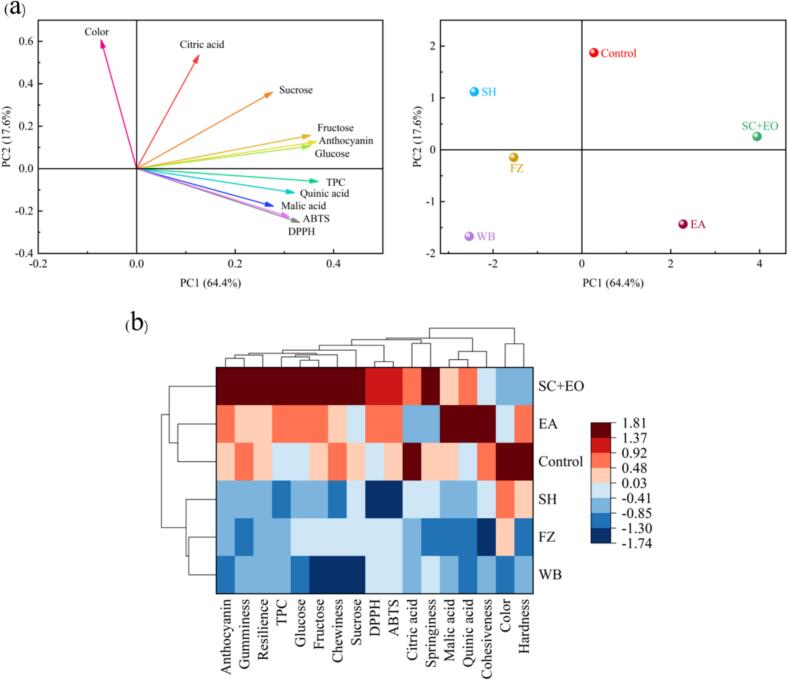
Principal Component Analysis and Clustering Analysis of cherries under different pretreatment conditions.

To comprehensively assess the impact of various pretreatment methods on the quality of dried cherries, both physicochemical properties and sensory attributes were analyzed using PCA and CA. As shown in Fig. 9(a), the PCA results indicate that PC1 and PC2 cumulatively explain 82 % of the variance. Among them, PC1 contributed 64.4 % and was positively correlated with changes in physicochemical parameters such as glucose, fructose, sucrose, anthocyanin, and TPC. PC2, which explains 17.6 % of the variance, is primarily influenced by color and citric acid content. SC + EO pretreatment achieved the highest score along the PC1 axis, demonstrating significant benefits in terms of antioxidant activity, TPC, anthocyanin retention, and sugar preservation. In contrast, the control group exhibited higher scores along the PC2 axis, correlating with color and citric acid content. The WB and SH groups were located far from the origin, indicating that their quality deviated from the ideal state. The clustering heat map analysis (Fig. 9(b)) revealed significant differences in quality indicators and sensory attributes among the pretreatment methods. SC + EO, Control, and EA pretreated dried cherries were grouped together, indicating a certain degree of similarity in terms of quality retention. SH, FZ, and WB were grouped together because they showed a consistent downward trend in quality. Compared to the other groups, the SC + EO group exhibited significantly higher levels of anthocyanin, springiness, chewiness, TPC, and antioxidant activity. While the control and EA treatments showed similar trends across most indices, their overall performance was inferior to SC + EO. The SH and WB groups had lower scores for color, hardness and organic acid content, and showed more obvious quality deterioration. Notably, the WB group recorded the lowest values across the majority of critical quality parameters. Comprehensive analysis shows that the results of PCA and CA are highly consistent, both indicating that SC + EO pretreatment can improve the physicochemical quality and sensory attributes of dried cherries.

## 4 Conclusion

This study systematically investigated the effects of five pretreatment methods (SC + EO, EA, WB, FZ, and SH) on USA-HAD dried cherries, evaluating them across a spectrum of factors, including drying efficiency, energy consumption, physicochemical quality, microstructure, and sensory characteristics. The findings revealed that all pretreatments accelerated the dehydration process, significantly reducing drying time (by 16.67 % to 38.89 %) and energy consumption (by 21.25 % to 47.17 %). Among the various treatments, SC + EO demonstrated the most substantial benefits, notably enhancing the retention of anthocyanins, TPC, individual sugars, as well as malic and quinic acids. Furthermore, SC + EO pretreatment significantly improved antioxidant activity (DPPH and ABTS assays), texture attributes, and overall sensory acceptance. The WB pretreatment emerged as the most energy-efficient method, consuming only 114.76 kWh/kg, while also maintaining color stability (ΔE = 1.87 ± 0.14). Microstructural analysis further supported the efficacy of SC + EO, showing the formation of microporous channels and surface cracks, which facilitated enhanced water diffusion. This structural modification is attributed to the breakdown of the wax and cuticle layers in an alkaline oleic acid ethyl ester environment, promoting the migration of both water and active compounds. PCA and CA analyses consistently underscored the superiority of SC + EO pretreatment, demonstrating that the physicochemical and sensory properties of these cherries most closely resemble those of fresh fruit.

Compared with existing studies, this research confirms the advantages of USA drying technology in enhancing moisture diffusion rates and preserving active ingredients. It also reveals variations in the synergistic effects of different pretreatment methods when combined with ultrasonication. Furthermore, in contrast to previous reports on edible coatings coupled with US or microwave pretreatment of cherries, this study systematically evaluates the impact of chemical, physical and thermal pretreatments on USA-HAD dried cherries. It clearly demonstrates the comprehensive benefits of SC + EO pretreatment in improving drying efficiency and product quality.

In summary, the SC + EO pretreatment combined with USA-HAD technology shows great promise in improving the efficiency of cherry drying, enhancing physicochemical quality and optimising sensory attributes. This study not only provides theoretical and practical foundations for optimising cherry drying processes, but also offers a reference pathway for high-quality drying processing of other small berry fruits.

## CRediT authorship contribution statement

**Hongyang Lu:** Writing – review & editing, Writing – original draft, Visualization, Validation, Software, Methodology, Formal analysis, Data curation, Conceptualization. **Guojun Ma:** Validation, Resources, Methodology, Investigation. **Fangxin Wan:** Validation, Resources, Project administration, Funding acquisition. **Zepeng Zang:** Methodology, Investigation. **Yanrui Xu:** Methodology, Investigation. **Bowen Wu:** Methodology, Investigation. **Lingli Li:** Methodology, Investigation. **Zelin Liu:** Methodology, Investigation. **Xiaopeng Huang:** Validation, Supervision, Resources, Project administration, Funding acquisition, Formal analysis. **Fei Dai:** Validation, Resources, Methodology.

## Declaration of competing interest

The authors declare that they have no known competing financial interests or personal relationships that could have appeared to influence the work reported in this paper.
